# Microwave and Antenna Systems in Medical Applications

**DOI:** 10.3390/s24041059

**Published:** 2024-02-06

**Authors:** Hoi-Shun Lui, Mikael Persson

**Affiliations:** 1 College of Sciences and Engineering, University of Tasmania, Sandy Bay, TAS 7005, Australia; 2 Division of Signal Processing and Biomedical Engineering, Department of Electrical Engineering, Chalmers University of Technology, SE-41296 Gothenburg, Sweden

The non-ionizing nature of microwave radiation and the low cost of microwave electronics offer innovative solutions for medical diagnosis, treatment, and health monitoring [1,2]. Researchers in antennas, microwave electronics, computational electromagnetics, imaging, and signal processing are working collaboratively with medical practitioners to enhance our lives by developing next-generation healthcare technologies. A well-known example would be the prospect of utilizing microwave sensors for stroke detection such that prehospital diagnosis can be facilitated in ambulances using a portable system [3,4,5]. In regional areas in Sweden and Australia, stroke patients and the elderly who are located remotely from hospitals will benefit. Clinical trials of microwave stroke detection and breast screening are currently in place across Europe, North America, and Oceania. Another example would be the use of microwave radiation for breast screening, providing alternatives to existing ionizing X-ray mammography for cancer diagnosis [6,7,8]. 

The recent boom in artificial intelligence (AI) and machine learning (ML) is opening new avenues that accelerate technical development in microwave-based techniques for medical applications. These developments cover signal processing, imaging processing, as well as microwave and antenna system optimization [9,10].

In this Special Issue, we present a comprehensive exploration of microwave and antenna systems, showcasing their transformative impact on diverse medical applications, including medical diagnosis, treatment, and patient monitoring. This collection features nine cutting-edge original research articles followed by two insightful reviews incorporating microwave imaging and sensing into digital pulmonology and digital gastroenterology. The titles and authors of the articles are listed in Table 1.

## 1. Original Research Articles

**Medical Diagnosis:** There are three articles (i–iii) that deal with the use of microwave imaging and sensing for medical diagnosis of breast cancer, hamstring muscle injuries, and stroke detection. Sekehravani and Leone et al. (i) address the inverse scattering problem for dielectric cylinders, providing insights into achievable resolution for medical imaging and highlighting the potential impact of microwave breast cancer imaging on cancer diagnosis. In (ii), researchers from Sweden propose a semicircular microwave imaging array with a novel antenna design for imaging hamstring muscle injuries. The inclusion of a lossy gel in the imaging domain proves instrumental in reducing multipath signals, enhancing imaging quality for more accurate diagnostics. Pokorny et al. (iii) investigate microwave brain stroke detection and classification using support vector machines, emphasizing the importance of training data in achieving high accuracy. The study showcases the potential for microwave technology in diagnosing cerebral conditions.

**Disease Treatment:** There are three articles about microwave cancer treatment using microwave hyperthermia (iv) and microwave ablation (v,vi). Yildiz et al. (iv) evaluate the effectiveness of different fractal octagonal ring antenna designs. Their research emphasizes the critical role of applicator design in optimizing the specific absorption rate for breast tumor hyperthermia applications. Examining microwave ablation in bone tumors, Trujillo-Romero et al. (v) evaluate the thermal performance of multi-antenna arrays. Using the finite element method, their study presents various antenna configurations, demonstrating the feasibility of treating bone tumors with a specific focus on ablated tissue volume. Frandon et al. (vi) investigate the early response and reliability in treating liver, kidney, and lung lesions using a commercial microwave ablation system in a clinical setting. Their findings underscore the system’s potential in patient monitoring through reliable ablations.

**Patient Monitoring**: There are two papers on patient monitoring using antenna systems (vii,viii), and one paper on implantable antenna design for brain–machine communication applications (ix). Zeng et al. (vii) utilize millimeter-wave radar for gait analysis in a real-life environment. Their research showcases the method’s reliability in measuring step time, offering potential applications in fall prevention for the elderly. In (viii), a flexible sinusoidal-shaped antenna sensor is proposed, and its application in strain sensing is explored. Their study demonstrates improved sensitivity and flexibility for wearable sensors, opening avenues for patient monitoring in various medical scenarios. Sapari et al. (ix) introduce an end-fire radiating implantable antenna for brain–machine interfaces; this research emphasizes high-data-rate wireless communication. Their study highlights the antenna’s enhanced gain and broadband operation (3 to 5 GHz), showcasing the potential of implanting antennas in the skull during brain surgery.

## 2. Review Articles

This Special Issue includes two review articles on the potential integration of microwave sensing into the medical diagnosis of respiratory disorders (x) and digestive system disorders (xi). The review in (x) delves into the integration of AI with dual microwave acoustic sensing and imaging for the analysis of lung sounds. The exploration of AI models opens avenues for real-time respiratory sound analysis, potentially revolutionizing clinical pulmonology practice. The review in [xi] focuses on digital gastroenterology, investigating the potential of a bowel sound recording and analysis device—the phonoenterogram. With a spotlight on microwave-based digital phonoenterography, the review envisions a future where AI-driven analysis of bowel sounds becomes an accessible, cost-effective, and versatile diagnostic tool.

## 3. Conclusions

This Special Issue highlights the multifaceted contributions of microwave and antenna systems to revolutionizing healthcare. The original research presents innovative approaches to various technical challenges, showcasing the potential of microwave technology in diverse applications. The review papers pave the way for integrating microwave sensing and imaging solutions, opening avenues for novel diagnostic applications.

## Figures and Tables

**Table 1:** List of articles included in this Special Issue.

	Title	Authors
(i)	Evaluation of the Resolution in Inverse Scattering of Dielectric Cylinders for Medical Applications	Ehsan Akbari Sekehravani, Giovanni Leone
(ii)	Microwave Antenna System for Muscle Rupture Imaging with a Lossy Gel to Reduce Multipath Interference	Laura Guerrero Orozco, Lars Peterson, Andreas Fhager
(iii)	On the Role of Training Data for SVM-Based Microwave Brain Stroke Detection and Classification	Tomas Pokorny, Jan Vrba, Ondrej Fiser, David Vrba, Tomas Drizdal, Marek Novak, Luca Tosi, Alessandro Polo, Marco Salucci
(iv)	Comparison of Microwave Hyperthermia Applicator Designs with Fora Dipole and Connected Array	Gulsah Yildiz, Iman Farhat, Lourdes Farrugia, Julian Bonello, Kristian Zarb-Adami, Charles V. Sammut, Tuba Yilmaz, Ibrahim Akduman
(v)	Thermal Evaluation of Multi-Antenna Systems Proposed to Treat Bone Tumors: Finite Element Analysis	Citlalli Jessica Trujillo-Romero, Juan Dionisio Merida, Texar Javier Ramírez-Guzmán, Raquel Martínez-Valdez, Lorenzo Leija-Salas, Arturo Vera-Hernández, Genaro Rico-Martínez, José Jesús Agustín Flores-Cuautle, Josefina Gutiérrez-Martínez, Emilio Sacristán-Rock
(vi)	Microwave Ablation of Liver, Kidney, and Lung Lesions: One-Month Response and Manufacturer’s Charts’ Reliability in Clinical Practice	Julien Frandon, Philippe Akessoul, Tarek Kammoun, Djamel Dabli, Hélène de Forges, Jean-Paul Beregi, Joël Greffier
(vii)	Walking Step Monitoring with a Millimeter-Wave Radar in Real-Life Environment for Disease and Fall Prevention for the Elderly	Xuezhi Zeng, Halldór Stefán Laxdal Báruson, Alexander Sundvall
(viii)	Wearable Sensor Based on Flexible Sinusoidal Antenna for Strain Sensing Applications	Mehran Ahadi, Mourad Roudjane, Marc-André Dugas, Amine Miled, Younès Messaddeq:
(ix)	Brain Implantable End-Fire Antenna with Enhanced Gain and Bandwidth	Lisa Sapari, Samnang Hout, and Jae-Young Chung
(x)	Digital Pulmonology Practice with Phonopulmography Leveraging Artificial Intelligence: Future Perspectives Using Dual Microwave Acoustic Sensing and Imaging	Arshia K. Sethi, Pratyusha Muddaloor, Priyanka Anvekar, Joshika Agarwal, Anmol Mohan, Mansunderbir Singh, Keerthy Gopalakrishnan, Ashima Yadav, Aakriti Adhikari, Devanshi Damani, Kanchan Kulkarni, Christopher A. Aakre, Alexander J. Ryu, Vivek N. Iyer, Shivaram P. Arunachalam
(xi)	Practicing Digital Gastroenterology through Phonoenterography Leveraging Artificial Intelligence: Future Perspectives Using Microwave Systems	Renisha Redij, Avneet Kaur, Pratyusha Muddaloor, Arshia K. Sethi, Keirthana Aedma, Anjali Rajagopal, Keerthy Gopalakrishnan, Ashima Yadav, Devanshi N. Damani, Victor G. Chedid, Xiao Jing Wang, Christopher A. Aakre, Alexander J. Ryu, Shivaram P. Arunachalam
